# Rare earth composite MOFs materials for energy, environmental and medical applications

**DOI:** 10.1039/d5ra02759d

**Published:** 2025-06-24

**Authors:** Haonan Pei, Guilin He, Shuaibo Jiang, Bingxin Xue, Fangdi Huang, Hongqun Tang, Nannan Wang, Yanqiu Zhu

**Affiliations:** a State Key Laboratory of Featured Metal Materials and Life-cycle Safety for Composite Structures, MOE Key Laboratory of New Processing Technology for Nonferrous Metals and Materials, School of Resources, Environment and Materials, Guangxi University Nanning 530004 China wangnannan@gxu.edu.cn; b Faculty of Environment, Science and Economy, University of Exeter EX4 4QF UK Y.zhu@exeter.ac.uk; c Key Laboratory of High Performance Structural Materials and Thermo-surface Processing (Guangxi University), Education Department of Guangxi Zhuang Autonomous Region Nanning 530004 China

## Abstract

Rare Earth Elements (REEs) are a group of seventeen specialized elements that have shown irreplaceable roles in energy, environment and medical fields due to their unique electronic structures and chemical properties. Rare Earth Composite Metal–Organic Frameworks (RE-MOFs) have become a hotspot for interdisciplinary research as they combine the excellent properties of REEs with the porous properties of MOFs, which have high specific surface area, adjustable pore size, abundant active sites, and multifunctionality. In this paper, we systematically review the progress of the application of RE-MOFs in the fields of energy storage and conversion, pollution control, and biomedicine, analyze the performance optimization strategies and challenges, and look forward to the future development direction. Through integrated material design, innovation in synthesis methods and interdisciplinary cooperation, RE-MOFs are expected to drive the commercialization of next-generation high-performance materials and help achieve the global sustainable development goals.

## Abstract 2

Rare Earth Elements (REEs) are a group of seventeen specialized elements that have shown irreplaceable roles in energy, environment and medical fields due to their unique electronic structures and chemical properties.

## Graphical abstract

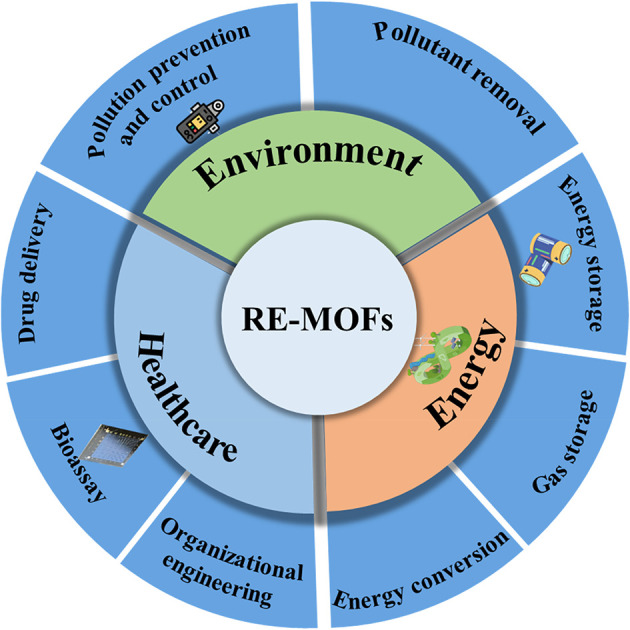

## 1. Introduction

Rare earth elements (REEs) are known as the “vitamins of modern industry” because of their unique 4f electron layer structure and variable oxidation states, which have shown irreplaceable functionality in the fields of catalysis, optics, magnetism and energy conversion. With the growing global demand for clean energy,^1,2^ environmental governance and precision medicine,^3,4^ the boundaries of rare earth element applications continue to expand. Meanwhile, metal–organic frameworks, as a class of porous crystalline materials formed by the self-assembly of metal nodes and organic ligands, have become a research hotspot in the field of materials science by virtue of their high specific surface area, adjustable pore size, abundant active sites, and multifunctionality.^5–8^ In the environmental field, MOFs show great promise as desalination membrane materials for desalination of seawater by virtue of their structural diversity, tunability, and porous voids providing secondary water channels.^9^ Furthermore, the selection of linkers and metal nodes along with the post-synthesis modifications to tune the pore-functionalized MOFs can also achieve efficient target-specific identification of aquatic inorganic pollutants.^10^ In the biomedical field, MOFs are an emerging candidate to mitigate environmental pollution and health diseases related to it.^11^

In recent years, the research on the application of RE-MOFs in the fields of energy, environment and medicine has made remarkable progress. In the field of energy, RE-MOFs have significantly improved the electrochemical energy storage efficiency and photoelectrocatalytic activity through the synergistic effect of rare earth ions and organic ligands. Examples include the high capacity properties of rare earth-doped MOFs for lithium-ion batteries,^12^ and photocatalytic CO_2_ reduction with a high selectivity.^13^ In environmental governance, the multistage pore and selective adsorption capabilities of RE-MOFs make them an efficient platform for pollutant detection and removal, such as fluorescent sensing of volatile organic compounds (VOCs),^14^ targeted adsorption of heavy metal ions and photocatalytic degradation of organic pollutants. In the biomedical field, RE-MOFs, with their luminescent properties, biocompatibility and drug loading capacity, have promoted the development of targeted drug delivery, multimodal imaging and intelligent diagnostic and therapeutic systems, for example, europium (Eu) MOFs-based fluorescent probes in the early diagnosis of cancer.^15^ Aptamer-guided nanocarriers with mesoporous MOF shells and upconverted luminescent NaYF_4_:Yb^3+^/Er^3+^ NPs for targeted drug delivery and cellular imaging.^16^

However, the large-scale application of RE-MOFs still faces many challenges. First, the cycling performance of the materials needs to be improved; second, the potential migration risk of rare earth ions and environmental safety need to be systematically evaluated;^13^ in addition, the problems of high synthesis cost and complex process^17^ also restrict their industrialization. To address these issues, the researchers have developed a new approach through topological modification,^18^ defect engineering,^19^ cross-material composites, and other strategies to optimize performance, to advance RE-MOFs from laboratory exploration to practical applications.

In this paper, we systematically review the latest research progress of RE-MOFs in the fields of energy storage and conversion, environmental remediation, and biomedicine, with a focus on analyzing the mechanism of performance optimization, and looking forward to the future development direction. Through the integration of material design, synthesis method innovation and multidisciplinary cross-collaboration, RE-MOFs are expected to provide transformative solutions to global challenges such as carbon neutrality, pollution control and precision medicine, and contribute to the realization of sustainable development goals.

## 2. Material design

The design and synthesis of novel functional materials is a common goal for researchers in the fields of science, engineering, and technology. MOFs have gained notable attention as potential functional materials due to their highly tunable synthesis. The introduced reticulation chemistry, which is the basis of MOF synthesis, is defined as the design and formation of ordered network materials with predetermined structures. This means that by understanding the geometry and connectivity of the building blocks, it is possible to build the same structure (or network) with several building blocks in order to generate a specific network structure.

Regardless of size, these building blocks can be manipulated to have the same (or similar) geometry and connectivity. These building blocks are inorganic metal nodes (ions, chains, or clusters) and organic connectors that come together to form secondary structural units (SBUs), which are assembled to form the framework material.^20^ Combining the unique properties of rare earth elements with the structural advantages of MOFs to form Rare Earth Composite Metal Organic Frameworks (RE-MOFs) not only inherits tunable porous structure^21^ and high adsorption capacity of MOFs, but also endows the materials with a new functional dimension through the optical, electrical, and magnetic properties of rare earth ions, which provides an important interdisciplinary innovation carrier.

The following is a systematic classification summary based on the types, doping mechanisms and functionalization applications of rare earth metal–organic frameworks (RE-MOFs), which provides theoretical references and practical guidelines for the rational design and cross-field applications of novel functional materials (Table 1).

**Table 1:** Doping mechanisms and applications of different types of RE-MOFs

RE-MOF type	Rare earth doping mechanism	Typical application
Ce-MOF	*In situ* coordination substitution: Ce^3+^/Ce^4+^ directly acts as a metal node, forming strong coordination bonds with carboxylic acid ligands (Ce–O bond energies >500 kJ mol^−1^), replacing conventional transition metal nodes	Photocatalytic degradation of pollutants, CO_2_ reduction
Eu-MOF	Ligand-sensitized luminescence: Eu^3+^ captures the energy of the ligand excited state through the “antenna effect” (ligand → Eu^3+^ energy transfer efficiency >80%), achieving efficient fluorescence emission	Fluorescence sensing (Hg^2+^ detection)
Tb-MOF	Rigid coordination locking: Tb^3+^ forms an octa-coordinated structure with a polydentate carboxylic acid ligand, which inhibits ion leakage through spatial site resistance (Tb^3+^ release rate <0.01%)	Biological microenvironment temperature monitoring
Gd-MOF	Rapid nucleation dominance: microwave heating accelerates the coordination kinetics of Gd^3+^ with carboxylic acid ligands, preferentially forming Gd–O clusters as stable SBUs (secondary structural units)	MRI contrast agent
Yb-MOF	Pore-limited domain ion exchange: Yb^3+^ enters the ZIF-8 pore by diffusion, displacing part of the Zn^2+^ site (exchange rate ∼30%) to form a Yb–N coordination bond	Tumor diagnosis
Nd-MOF	Macrocyclic ligand chelation: the four pyrrole nitrogens of the porphyrin form a planar tetragonal coordination with Nd^3+^, with a central cavity stabilizing Nd^3+^ (coordination constant log *K* > 15)	Photothermal therapy
Er-MOF	Dynamic coordination regulation: Er^3+^ forms a reversible coordination bond with a flexible fumarate ligand, and humidity change triggers the ligand to rotate, realizing pore switching (pore size change 0.3 → 1.2 nm)	Real-time monitoring of environmental humidity

## 3. Applications in the energy sector

### 3.1 Energy storage

Metal–organic frameworks (MOFs) represent a distinct type of hybrid materials, combining high porosities with diverse properties that arise from their organic and inorganic building units,^22^ with excellent properties such as morphological diversity, high porosity, large specific surface area, and abundant active sites,^23^ and thus show significant potential for application in aerospace^24^ and electrochemical energy storage. However, pristine MOF severely limits its application in electrochemical energy storage due to poor electrical conductivity and low specific capacitance.^25^ Rare earth elements (REEs) are widely used in energy storage due to their atomic structure with 4f electron layers and abundant electron energy levels, which can be combined with MOF structures to form a variety of new composites with unique properties. In this section, we summarize the application of RE-MOF materials to batteries and supercapacitors.

#### 3.1.1 Lithium ion battery

Lithium-ion batteries (LIBs) are widely favored for their high energy density, high voltage, low self-discharge and environmental friendliness. Graphite, as a widely used electrode material for LIBs, has gradually failed to meet the demand for high-capacity LIBs.^26^ In addition, it has the problem of lithium dendrites caused by low discharge potential, which seriously affects the safety of lithium-ion batteries.^27^ Therefore, scientists have developed various materials as electrode materials for LIB, such as carbon materials,^28,29^ metal oxides,^30,31^ metal sulfides,^32^ and other materials.

**Fig. 1:**
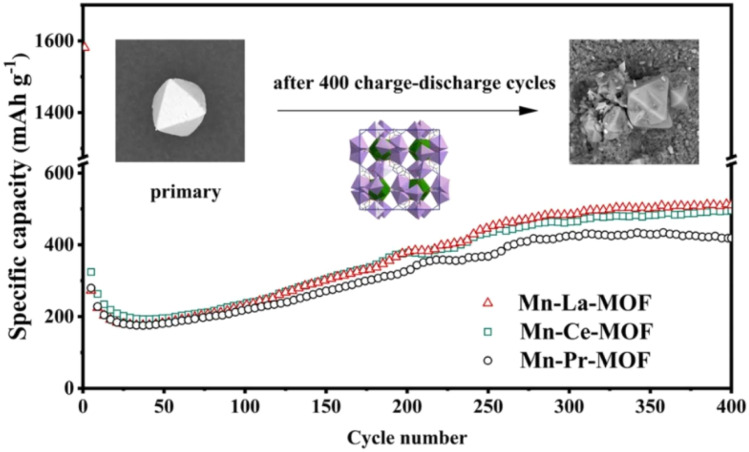
Current density of Mn–La MOF after 400 cycles. Reproduced from ref. 12 with permission from Elsevier, copyright 2022.

**Fig. 2:**
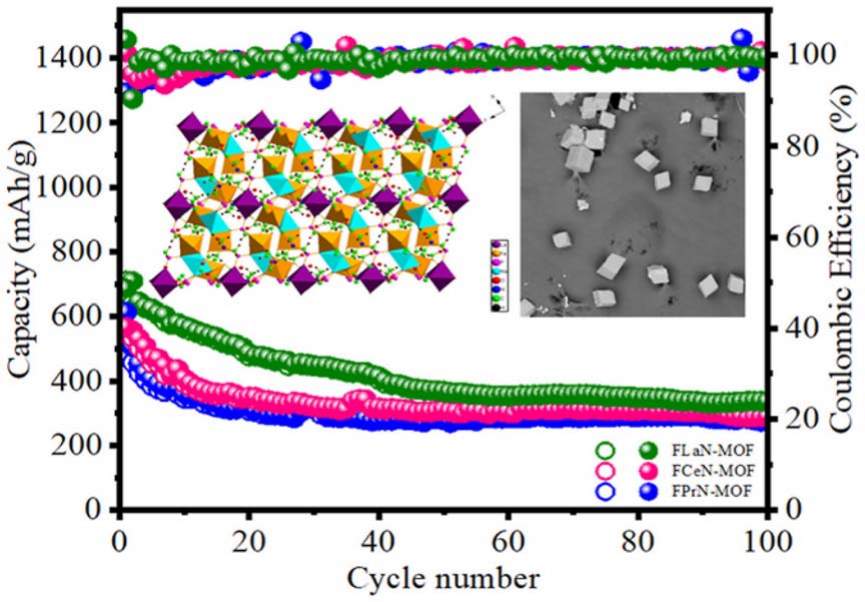
Reversible capacity of FLaN-MOF after 100 cycles at 100 mA g^−1^. Reproduced from ref. 35 with permission from Elsevier, copyright 2021.

The RE-MOFs materials can provide rich interfaces and efficient mass transfer channels for the electrochemical reactions inside the battery. The introduction of lanthanides, which have high charge density and strong coordination ability, can optimize the electronic structure and pore environment of MOFs, enhance electrostatic interactions with Li^+^ and selective adsorption, and thus improve the lithium ion storage performance. Therefore, RE-MOFs materials can be a candidate for solving the LIB electrode material problem. Zhao *et al.* synthesized a series of bimetallic metal–organic frameworks (MOFs) with the same spatial structure using 3,5-pyrazole dicarboxylic acid monohydrate as the organic linker.^33^ Mn–La MOF doped with lanthanides as an anode material for lithium-ion batteries (LIBs) exhibited the highest capacity (510.67 mAh g^−1^ after 400 cycles at 100 mA g^−1^ current density) and the lowest difficulty in ion transport, as shown in Fig. 1.^12^ Lv *et al.* prepared a new highly symmetric MOF material with the chemical formula C_30_H_40_Fe_6_LnN_6_Na_2_O_44_P_6_ (where Ln stands for lanthanum, cerium, and praseodymium) by a new ionothermal synthesis method.^34^ FLaN-MOF and commercial LiCoO_2_ were used as negative and positive electrodes, respectively, to assemble the cell for LIBs. The cell provides a reversible capacity of 145 mAh g^−1^ with a coulombic efficiency of 98.6%. After 100 cycles, the reversible specific capacity was as high as 337 mAh g^−1^, see Fig. 2.^35^ Tb-MOF, a rare earth complex, was successfully prepared by solvothermal method by Xia *et al.* Experiments have shown that the flexible skeleton of Tb-MOF provides efficient channels for lithium ion de-embedding, the abundant carboxyl groups and benzene rings construct chemisorption sites, and the strong Tb–O bond guarantees the reversibility of the electrode structure, which results in the excellent cycling stability of the complex.^36^

#### 3.1.2 Lithium–sulfur battery

**Fig. 3:**
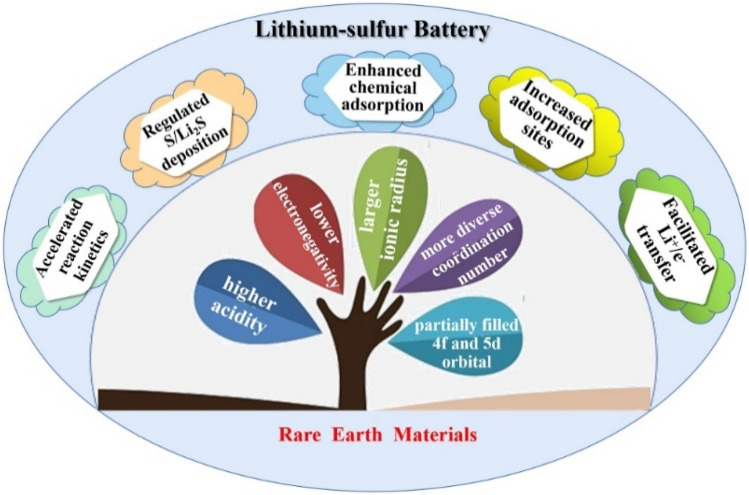
Rare earth compounds in lithium–sulfur batteries. Reproduced from ref. 40 with permission from Elsevier, copyright 2023.

Lithium–sulfur batteries (LSBs) are highly promising for future development due to their high theoretical specific capacity, high energy density, and cost-effectiveness. However, existing technologies suffer from low sulfur utilization due to the non-conductivity of elemental sulfur and its discharge products Li_2_S and Li_2_S_2_,^37^ collapse and pulverization of the electrode structure due to volume expansion,^38^ rapid depletion of active materials due to the “shuttle effect” of lithium polysulfides (LiPSs)^39^ and corrosion of lithium anode increased battery polarization, *etc.* The main problem is the shuttle effect caused by polysulfides. As a key part of lithium–sulfur battery system, the modification of the diaphragm can effectively alleviate the shuttle effect caused by soluble intermediates in LSBs, thus enhancing the comprehensive performance of LSBs and promoting the process of large-scale commercialization. In this regard, scientists have tried to modify the diaphragm by introducing rare earth elements (such as yttrium, samarium, *etc.*) into the MOFs material to improve the cycling performance and multiplication performance of LSBs, as shown in Fig. 3.^40^

The rare earth element yttrium has unique optical, electrical, magnetic and catalytic properties,^41^ and the composite of yttrium with MOF can endow the material with high specific surface area and excellent catalytic properties. Qian *et al.* prepared Y_2_O_3_–C@CNT composite diaphragm material by growing Y_2_O_3_–C nanoparticles *in situ* on the surface of carbon nanotubes *via* aqueous method, and then prepared Y_2_O_3_–C@CNT composite diaphragm material through high temperature carbonization. The experimental results show that the material is characterized by porous structure, high specific surface area, synergistic distribution of micro- and mesopores and excellent electrical conductivity, and the abundant polar sites on its surface can effectively anchor polysulfides. The PE diaphragm coated with this composite material enables the lithium–sulfur battery to obtain an initial discharge capacity of 900 mAh g^−1^ at 0.5C and 3 mg cm^−2^ high sulfur loading, and the capacity retention rate reaches 53.7% (483.85 mAh g^−1^) after 400 cycles, which is a significant en hancement of the cycling performance for LSBs.

**Fig. 4:**
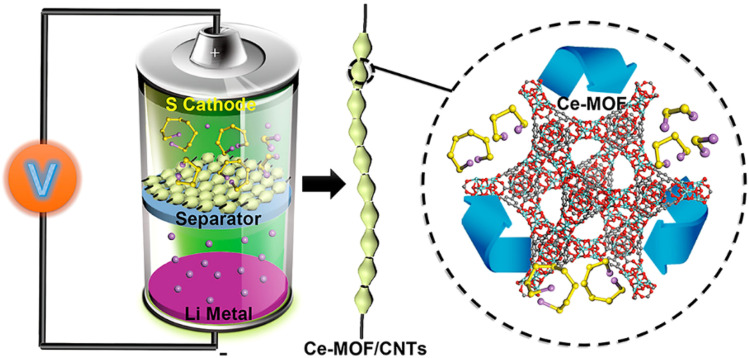
Ce-MOF-2/CNT as a diaphragm coating material for Li–S batteries. Reproduced from ref. 43 with permission from ACS Publications, copyright 2019.

CeO_2_ nanoparticles have received much attention due to their ability to adsorb and catalyze the transformation of polysulfides well,^42^ and combining CeO_2_ nanoparticles with MOF can rapidly adsorb sulfides and promote their rapid catalytic. Hong *et al.* synthesized MOFs containing Ce(iv)-cluster nodes, which were then combined with CNTs to form Ce-MOFs/CNT composites and used as the diaphragm coatings for LSBs.^43^ The experimental results show that the initial specific capacity of the Ce-MOF-2/CNT-coated cell is as high as 1021.8 mAh g^−1^, which slowly decreases to 838.8 mAh g^−1^ after 800 cycles, with a decay rate of 0.022% and a coulombic efficiency of nearly 100%. This demonstrated that the Ce-MOF-2/CNT porous diaphragm coating material (see Fig. 4 (ref. 43)) could play the dual functional roles of catalytic conversion of polysulfides and blocking of polysulfide transport pathways, thus effectively suppressing the shuttle effect in Li–S batteries. Liu *et al.* successfully synthesized phosphorus-doped porous CeO_2_ (P-CeO_2_) by solvothermal method, calcination and phosphatization treatment, and modified commercial PP diaphragm by coating process.^44^ The results show that at a sulfur loading of 1.28 mg cm^−2^, the cell capacity reaches 1180 mA h^−1^ with a decay rate of 0.10% per turn at 0.5C magnification. When the sulfur loading was increased to 2.0 mg cm^−2^, the decay rate per turn decreased to 0.048% at 0.5C multiplication. As a result, the P-CeO_2_ diaphragm promotes chemical uptake and catalytic conversion of polysulfides in lithium–sulfur batteries, leading to high performance and long cycle life.

In addition to the introduction of Y and Ce, Nd, as an active rare earth element, can be combined with MOF to have a unique pore structure and a large number of active sites, which can block the shuttle effect of LiPs and ensure the rapid penetration of the electrolyte and ion diffusion.^43^ Hao *et al.* synthesized Nd-MOF/KB and Nd-MOF/CNT materials by hydrothermal method and obtained Nd_2_O_3_–C/KB and Nd_2_O_3_–C/CNT composites by high-temperature carbonization treatment.^45^ The experimental results show that these carbonized composites have a porous structure and the metal oxides in them provide abundant adsorption sites, and these properties can effectively inhibit the migration of lithium polysulfides (LiPSs) and promote the redox transformations in the cathode reaction, and Nd_2_O_3_–C/KB exhibits better cycling and multiplication performance than Nd_2_O_3_–C/CNT when used as a battery separator performance.

#### 3.1.3 Supercapacitor

Supercapacitors (SCs), as a class of electrochemical energy storage devices with high energy density, can have capacities hundreds to thousands of times that of conventional electrolytic capacitors. The device also has the advantages of long cycle life and ecological compatibility.^46–48^ This makes it a new generation of energy storage technology with great potential for development. However, common electrode materials such as carbon, transition metal oxides, and conductive polymers require the development of new functional materials to facilitate the commercialization of supercapacitors due to their low capacitance, high cost, and poor stability.^49^

**Fig. 5:**
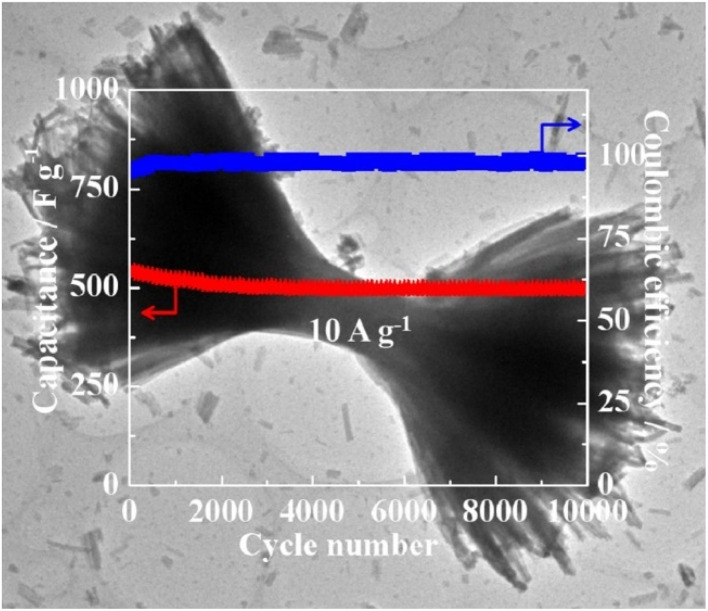
Capacitance of CeO_2_ electrode material after 10 000 cycles. Reproduced from ref. 52 with permission from Elsevier, copyright 2016.

CeO_2_, as an economical rare earth oxide with both environmental friendliness and excellent intrinsic redox activity,^50^ is regarded as a promising candidate for application, but its low specific surface area results in a significantly lower intrinsic theoretical specific capacity (∼560 F g^−1^) than that of transition metal oxides, which restricts its practical application in high-performance supercapacitors. MOF consists of metal ions or clusters and organic joints, which are compounds that can provide the highest surface area. Mutual bonding of CeO_2_ with MOF enhances its specific surface area and thus facilitates the commercialization of CeO_2_. Maiti *et al.* innovatively employed Ce-BTC metal–organic frameworks (cerium 1,3,5-benzenetricarboxylic acid ligand) as a sacrificial template, which were synthesized *via* solvothermal synthesis combined with a 650 °C/3 h calcination process to successfully prepare nanostructured CeO_2_ electrode materials.^51^ The experimental results show that 92% of the theoretical specific capacitance (based on the theoretical value of 560 F g^−1^) can be achieved in aqueous supercapacitors with this MOF-derived CeO_2_, and the pseudocapacitance performance occurs through the introduction of the K_4_Fe(CN)_6_/KOH composite electrolyte optimization strategy. The measured specific capacitance reaches 1204 F g^−1^, which exceeds the theoretical value by 115%. This dual-track strategy of “MOF template method + electrolyte engineering” provides a new idea to break the intrinsic capacitance limit of metal oxides. Zeng *et al.* developed a room-temperature alkali treatment method based on Ce-BTC MOF precursor, and successfully prepared CeO_2_ electrode materials with a graded dumbbell-like structure.^52^ The mesoporous template effect of the pristine MOF and the nanoparticle gap work together to give the CeO_2_ material a bimodal pore system that cuts through the mesopore and the microporous, which enhances the electrolyte wettability and ion diffusion rate. The experimental results show that this material exhibits a higher specific capacitance as a supercapacitor electrode when K_4_Fe(CN)_6_ is added to the KOH electrolyte. The maximum capacitance was 779 F g^−1^ at 1 A g^−1^, and the capacitance retention was close to 91% after 10 000 cycles, see Fig. 5.^52^

**Fig. 6:**
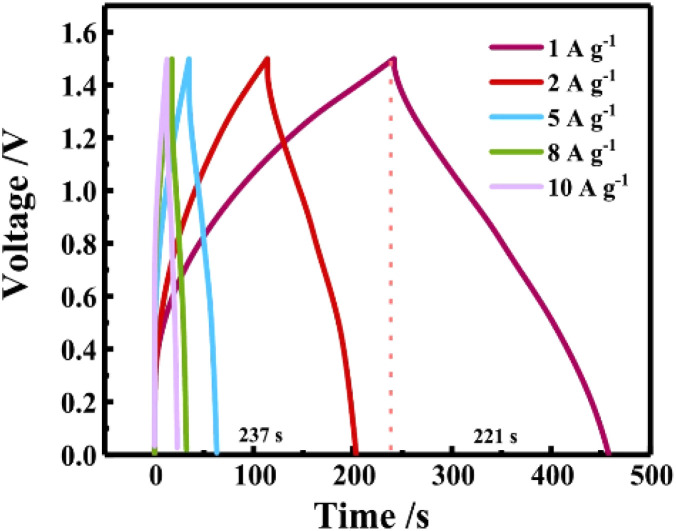
GCD curve for 1 to 10 A g^−1^. Reproduced from ref. 53 with permission from Elsevier, copyright 2024.

Hao *et al.* successfully prepared unique spherical-rod-like structure (NiCo)Se_2_-3 : 7-CeO_2_ materials by adjusting the ratio of ligands in the precursor MOF and using a one-step solvothermal method followed by a selenization process.^25^ The electrode material exhibits a specific capacitance of up to 2715 F g^−1^ (or 1222 mAh g^−1^) at a current density of 1 A g^−1^ and shows excellent multiplicative performance at 10 A g^−1^ with a loss of only 3.43% of the specific capacitance, solving the electrochemical energy storage and harvesting problem to some extent, see Fig. 6.^53^

Lanthanides other than Ce also have higher coordination numbers and flexible coordination geometries, which can increase the porosity and size of voids.^54^ Dezfuli *et al.* synthesized Eu-MOF by a hydrothermal method using fumarate and oxalate.^55^ Eu-MOF has a specific capacitance of 468 F g^−1^ discharge current density of 1 A g^−1^ and maintains 95.2% capacitance after 4000 cycles. Jafari *et al.* prepared by hydrothermal method [Tb_2_(fma)_2_(ox)(H_2_O)_4_·4H_2_O] rare earth metal–organic framework (Tb-MOF).^56^ The experimental results show that the Tb-MOF electrode exhibits a high specific capacitance of 510 F g^−1^ at a current density of 1 A g^−1^, and maintains a capacity retention of 351 F g^−1^ (68.8% capacity retention) even at a high current density of 16 A g^−1^, and a capacity retention of 91.9% after 4000 charge/discharge cycles. The capacity retention rate reaches 91.9% after 4000 charge/discharge cycles, providing a new paradigm for the design and application of rare earth MOFs in energy storage devices.

### 3.2 Gas storage

#### 3.2.1 Hydrogen storage

In response to the shortage of conventional energy sources, hydrogen has gained a lot of attention due to its excellent energy density. How to store hydrogen efficiently has become the focus of limiting the commercialization of hydrogen. Utilizing the high porosity property of rare earth composite MOF materials to store hydrogen has become the first choice to solve the hydrogen storage problem. Porous rare earth metal–organic frameworks Y(BTC)(H_2_O)·4.3H_2_O with high stability were synthesized by solvothermal reaction by Luo *et al.* It was shown that the samples exhibited permanent porosity, selective adsorption of hydrogen and good.^57^ At 77 K, the hydrogen adsorption isotherm was I-shaped, and the hydrogen uptake was about 2.1 wt% at saturation. When used as a hydrogen storage material it was able to achieve 3.7 wt% hydrogen storage at high hydrogen loading concentration, showing potential as a self-assembled nanostructure.

### 3.3 Energy conversion

#### 3.3.1 Hydrogen production from electrolytic water

Due to the unique pore structure of MOF, it provides more active sites for catalyst attachment and a conducting medium for electron transport.^58^ By synthesizing MOF materials with different configurations and embedding multifunctional groups on organic ligands, the catalytic activity can be enhanced without changing the MOF topology.^59,60^ In addition, rare earth elements can be doped into electrochemical catalysts to improve their performance by virtue of their high activity and excellent electronic structure. All in all, rare earth MOFs have higher coordination numbers and richer coordination geometries than transition metal ions as functional metal centers of MOFs. Since the 4f electron layer endows rare earth MOFs with special and electrical properties, they have the potential for application in electrocatalysis.^13^

Hydrogen energy is one of the world's important future energy sources, and hydrogen production from electrolyzed water has been the mainstream industrial hydrogen production technology.^61^ However, the problems of large overpotential and low electrochemical energy conversion efficiency during the hydrogen precipitation reaction^62^ have become the main obstacles to the commercialization of hydrogen energy, and the use of RE-MOFs materials can solve the above problems to a certain extent. Shi *et al.* prepared Er-MOF/NiS catalysts by loading NiS catalysts onto Er-MOF *via* a modified hot-solvent method.^63^ The results showed that the catalyst exhibited a significantly lower overpotential (115 mV), smaller Tafel slope (83.48 mV dec^−1^), smaller charge transfer resistance (11.304 Ω), and larger electrochemically active area (1432.025 cm^2^). Liao *et al.* applied the hot solvent method to prepare Er-MOF/MoS_2_ composites, which were obtained by using Er-MOF as a precursor and compounding MoS_2_.^61^ Compared to Er-MOF and MoS_2_ alone, the Er-MOF/MoS_2_ composite exhibits the best performance in terms of hydrolysis electrocatalytic activity. Specifically, the composite achieves a current density of 10 mA cm^−2^ at an overpotential of only 234 mV, while exhibiting excellent long-term durability, highlighting its superior catalytic performance.

**Fig. 7:**
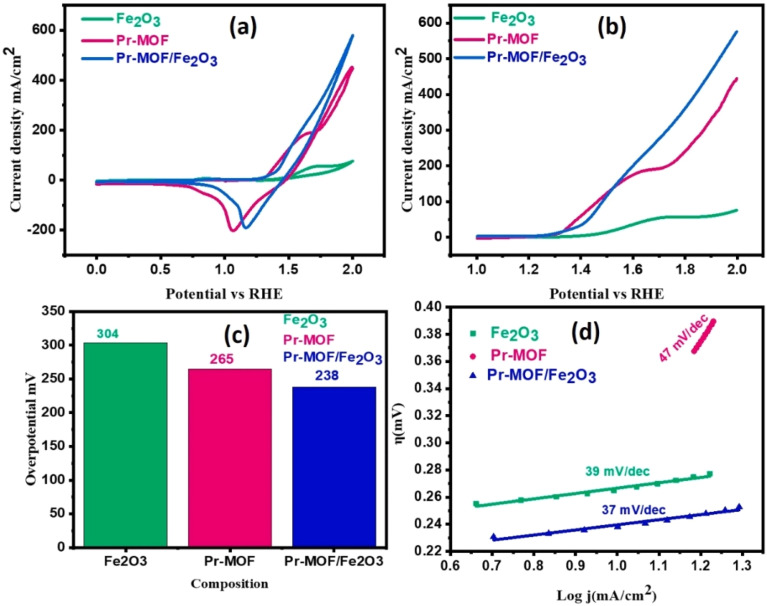
(a) Cyclic voltammograms; (b) linear scanning voltammograms; (c) comparison of overpotentials at a current density of 10 mA cm^−2^; and (d) Tafel slopes for Fe_2_O_3_, Pr-MOF, and Pr-MOF/Fe_2_O_3_. Reproduced from ref. 65 with permission from Elsevier, copyright 2023.

Another important reaction in hydrogen production from electrolyzed water is the Oxygen Evolution Reaction (OER). The use of rare-earth composite MOF materials as catalysts can improve the overall electrochemical energy conversion efficiency of electrolyzed water. Shabbir *et al.* successfully prepared innovative Pr-MOF/Fe_2_O_3_ composites by hydrothermal synthesis under controlled conditions. Pr-MOF/Fe_2_O_3_ nanocomposites at a current density of 10 mA cm^−2^ exhibited remarkable OER properties with low onset potential (1.41 V), low overpotential (238 mV) and low Tafel slope value (37 mV dec^−1^).^64^ In addition, the composite showed good long-term stability in 1 M alkaline solution (KOH) with no change in current density or performance. Fig. 7 (ref. 65) demonstrates the polarization curves of nanomaterials deposited on NF (nickel foam) in aqueous solution (1 M potassium hydroxide).

**Fig. 8:**
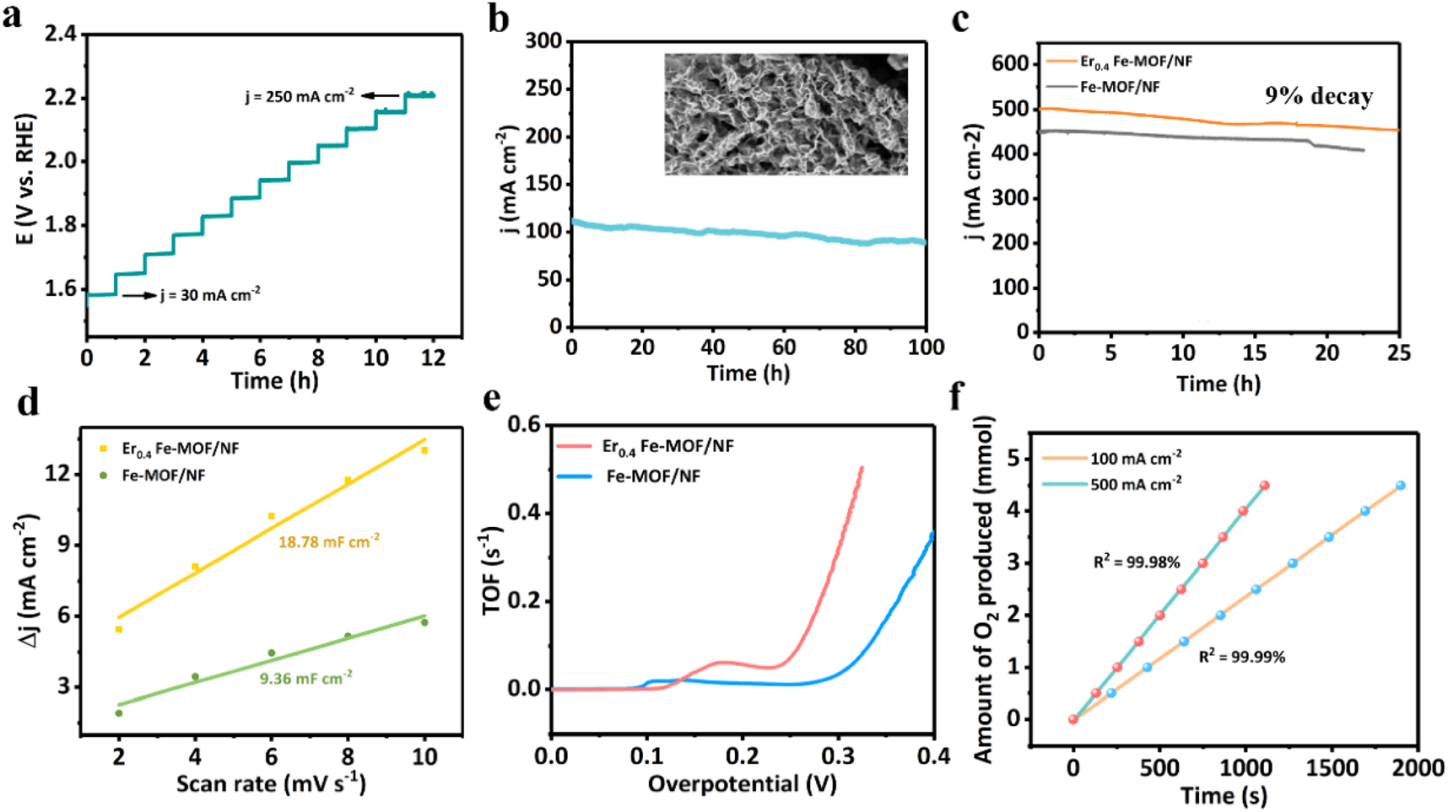
(a) Multi-current process curves of Er_0.4_Fe-MOF/NF (b) current densities of 30–250 mA cm^−2^ obtained at 3600 s intervals. Time-varying (*i*–*t*) curves at a current density of 100 mA cm^−2^, and the inset is a post-test SEM image. (c) Time-varying curves of Er_0.4_Fe-MOF/NF and Fe-MOF/NF. (e) ECSA evolution (d) and TOF values of Er_0.4_Fe-MOF/NF and Fe-MOF/NF. (f) Experimental amount of oxygen produced by the Er_0.4_Fe-MOF/NF electrode at 100 and 500 mA cm^−2^. Reproduced from ref. 67 with permission from MDPI, copyright 2021.

Ma *et al.* successfully prepared OER electrocatalysts with ultra-high activity and stability by one-step hot solvent method with optimized Er doping.^66^ Compared to the precursor Fe-MOF/NF, the Er_0.4_Fe-MOF/NF electrode excels in OER performance, being able to achieve a current density of 100 mA cm^−2^ at an overpotential of 248 mV and exhibiting a long-term electrochemical durability of at least 100 hours. In addition, the electrode is able to achieve high current densities of 500 mA cm^−2^ and 1000 mA cm^−2^ at very low overpotentials (297 mV and 326 mV, respectively). Fig. 8 (ref. 67) exhibits the electrochemical performance of Er_0.4_Fe-MOF/NF.

#### 3.3.2 Photocatalytic decomposition of water to produce hydrogen

In the field of photocatalysis, the porous structure and metal nodes of MOFs enable them to be used as carriers for semiconductor quantum dots, and the organic structure in MOFs can excite these quantum dots.^68^ In addition, according to the report of Sun *et al.*, the rare earth atoms can easily gain or lose electron to assist the charge transfer in the catalytic reaction because of their partly filled 4f orbit in electron structure.^69^ For example, the unstable valence state between Ce^3+^ and Ce^4+^, whose oxides can be used to catalyze some high value-added reactions. This unique feature allows for the generation of diverse luminescence transitions. Consequently, integrating rare earth elements into MOFs enhances their luminescent properties.^70^ Therefore, rare-earth MOFs are considered as potential photocatalysts.

**Fig. 9:**
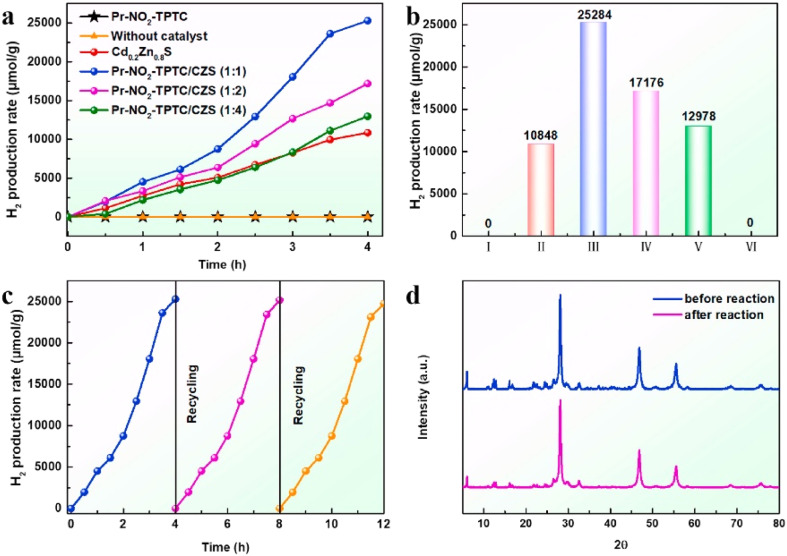
(a) and (b) Hydrogen production rate profiles of Pr–NO_2_–TPTC/CZS photocatalysts under full light conditions ((I) Pr–NO_2_–TPTC, (II) Cd_0.2_Zn_0.8_S, (III) Pr–NO_2_–TPTC/CZS (1 : 1), (VI) Pr–NO_2_–TPTC/CZS (1 : 2), (V) Pr–NO_2_–TPTC/CZS (1 : 4), (VI) no catalyst); (c) recovery performance of Pr–NO_2_–TPTC/CZS (1 : 1). (d) PXRD patterns of Pr–NO_2_–TPTC/CZS (1 : 1) before and after photocatalytic reaction. Reproduced from ref. 72 with permission from Elsevier, copyright 2024.

Hydrogen, as an efficient and clean energy medium, has the potential to be widely used as a green energy source, and photocatalytic hydrogen production is a potentially sustainable method of converting water to hydrogen using solar energy.^71^ For efficient photocatalytic hydrogen production, it is necessary to develop photocatalysts with excellent activity and stability. Li *et al.* successfully synthesized Pr–NO_2_–TPTC crystals based on binuclear Pr–O clusters by hot solvent reaction, and composited them with Cd_0.2_Zn_0.8_S at different mass ratios to prepare a series of stable Pr–NO_2_–TPTC/CZS composites.^72^ When the mass ratio of Pr–NO_2_–TPTC to Cd_0.2_Zn_0.8_S was 1 : 1, the composite material showed the best photocatalytic hydrogen production performance with a hydrogen production capacity of 6321 μmol g^−1^ h^−1^, which was higher than that of most reported CZS based heterojunction photocatalysts, and Fig. 9 (ref. 72) demonstrates the photocatalytic performance of this compliant material.

Sun *et al.* successfully synthesized a Gd-MOF based on a dye analog. Gd^3+^, as a rare earth metal ion, has a high coordination number and a strong coordination ability, and forms a stable coordination network with the polydentate ligands in the dye analogs, so that the MOF exhibited extremely high stability in different pH solutions.^73^ When 1.5% Ag was deposited as a co-catalyst on Gd-MOF, its photocatalytic activity was significantly increased to 1.5 times of the original Gd-MOF. Electrochemical Impedance Spectroscopy (EIS) and Photoluminescence (PL) tests also confirmed the fast transfer of photogenerated carriers and low complexation rate in Ag(1.5)/Gd-MOF. This rare-earth MOFs composite provides a new strategy for the construction of MOF photocatalysts for efficient solar hydrogen production.

#### 3.3.3 Carbon dioxide reduction

CO_2_ is a gas that can cause a greenhouse effect and has a direct role in today's global warming. Renewable solar energy is utilized to convert CO_2_ into high value-added products (*e.g.*, formic acid, acetic acid, and methane).^13^ CO_2_ emissions can be reduced and the goal of carbon neutrality can be achieved.

**Fig. 10:**
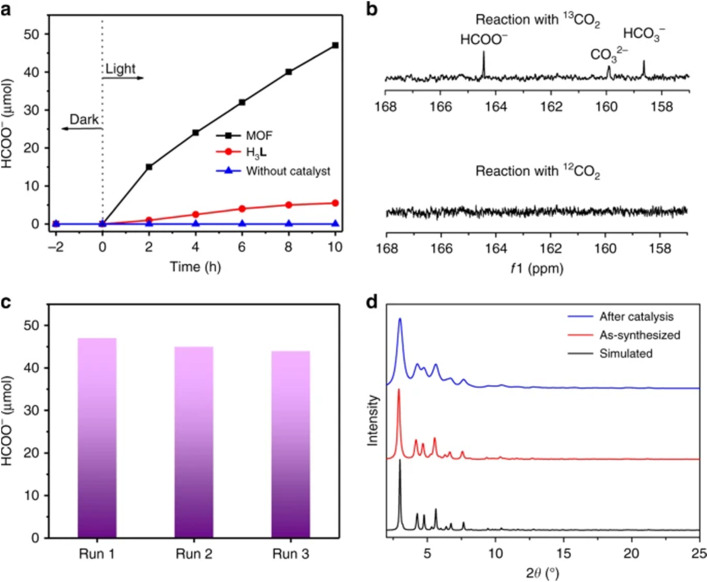
(a) Time profiles of HCOO^−^ production catalyzed by Eu–Ru(phen)_3_-MOF or H_3_L with or without catalyst under Xe lamp (420–800 nm) irradiation. (b) ^13^C NMR spectra of the liquid phase products after reaction with ^13^CO_2_ and ^12^CO_2_, respectively. (c) Amount of HCOO^−^ produced by three repetitions. (d) PXRD spectra of Eu–Ru(phen)_3_-MOF before synthesis and after photocatalytic reaction, showing that it maintains a good structure during the catalytic reaction. Reproduced from ref. 75 with permission from Springer Nature, copyright 2018.

MOFs show remarkable potential in the field of photocatalytic CO_2_ reduction reaction (CO_2_RR) due to their precisely designable metal–oxygen cluster nodes with topologically tunable multilevel pore systems. Meanwhile, rare-earth cations possess rich energy-level structures that can be combined with MOFs to significantly improve their catalytic efficiency. Therefore, it is particularly important to develop efficient catalysts that react under mild conditions, and rare-earth MOFs composites have received extensive attention due to their excellent physicochemical properties. Yan *et al.* prepared a triangular Ru(phen)_3_-derived tricarboxylic acid ligand (H_3_L) by a multistep reaction with Eu(NO_3_)_3_·6H_2_O and 2-fluorobenzoic acid (2-FBA) in dimethylformamide (DMF) to synthesize Eu–Ru(phen)_3_-MOF.^74^ Under visible light irradiation (420 nm < *λ* < 800 nm), the Eu–Ru(phen)_3_-MOF exhibited efficient CO_2_ reduction activity, with a formate generation rate of 321.9 μmol per h per mmol MOF, and Fig. 10 (ref. 75) demonstrated the photocatalytic performance. The catalytic properties of the catalytic materials Gd–TPTC–NH_2_ and Gd–TPTC–NH–[BMIM]Br in solvent-free catalytic cycloaddition reactions were investigated by Bao *et al.*^76^ It was found that Gd–TPTC–NH–[BMIM]Br exhibited excellent catalytic activity under optimized conditions, with yields up to 91% of 4-phenyl-1,3-dioxolan-2-one. The catalytic activity of the catalyst remained unchanged after five repeated uses, showing good stability and structural integrity.^76^ In addition, Gd–TPTC–NH–[BMIM]Br can effectively catalyze the cycloaddition reactions of other epoxides in yields as high as 95%, demonstrating its broad applicability in cycloaddition reactions with different substrates.

## 4. Applications in the environmental field

### 4.1 Pollution prevention and control

#### 4.1.1 VOCs detection

**Fig. 11:**
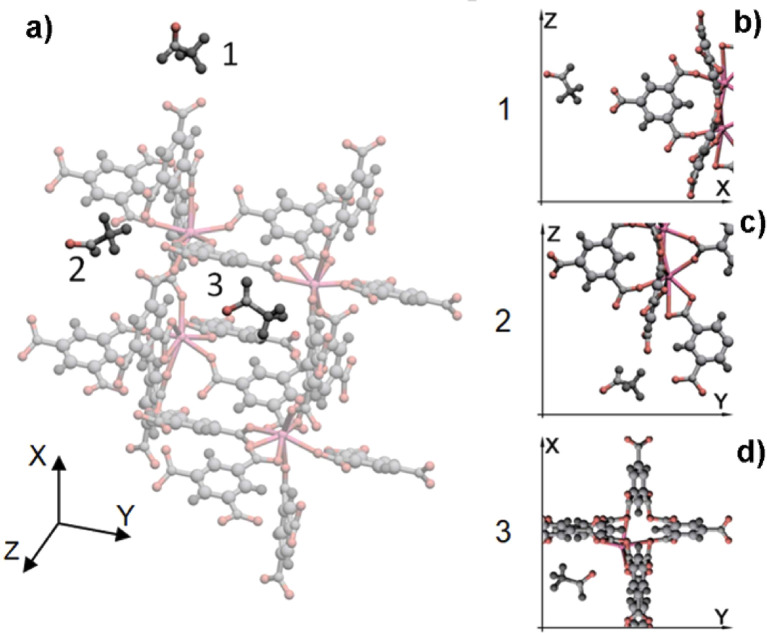
The adsorption process of acetaldehyde molecules in the La-BTC MOF passes through three states of the biomarker molecule (a): position 1 outside the MOF (a and b), position 2 close to the frame node (a and c), and position 3 inside the cage near the La^3+^ cation (a and d). Reproduced from ref. 79 with permission from Elsevier, copyright 2023.

Volatile organic compounds (VOCs) are present in the human exhaled gas mixture^77^ and have a negative impact on human health, predisposing to chronic and acute diseases.^78^ Microscopic mechanism of VOCs adsorption within the pores of La-BTC MOF revealed by molecular dynamics (MD) simulations by Gaidamavichute *et al.*^79^ The material exhibits ultra-high sensitivity for oxygen-containing biomarkers (*e.g.*, hexanoic acid, butyric acid, and acetone in volatile fatty acids), however, the detection efficiency for acetaldehyde molecules and hydrocarbons (ethane and neopentane) is low. The linear correlation between the polarizability and adsorption potential of biomarker molecules and the difference in spatial distances between the oxygen atoms (or other adsorption sites) in the adsorbed molecules and La^3+^ suggests that the high sensitivity of the La-BTC MOF to the molecules of oxygen-containing biomarkers in the pore space is mainly due to the adsorption governed by the strong electrostatic interactions between the oxygen atoms and the La^3+^ cations mechanism. For example, the three stages of adsorption of acetaldehyde molecules in La-BTC MOF are shown in Fig. 11.^79^

Among all volatile gaseous pollutants, formaldehyde is one of the most harmful gases,^80^ prolonged inhalation of formaldehyde can cause respiratory dysfunction, long-term exposure can lead to skin necrosis, liver damage, and even a variety of cancers.^81–84^ Zhu *et al.* successfully synthesized a new type of Eu-MOF by a solvothermal method, which exhibits a strong red fluorescence and is capable of detecting formaldehyde in both gas and liquid phases.^85^ This combination of sensitivity and multi-phase detection capability makes Eu-MOF show significant application value in the pollutant monitoring module of intelligent air purification systems.

#### 4.1.2 Transducers

Fine-tuning the internal pore size of the MOF enables selective recognition, uptake, and release of specific targets based on molecular size, and immobilized functional sites (*e.g.*, open metal sites or Lewis acid/basic sites) can be used as specific binding sites to enhance sensitivity through ligand or hydrogen-bonding interactions with target analytes. As a result, more and more fluorescent materials applied to the detection of various pollutants are being developed for the detection of metal ions, anions, volatile organic compounds (VOCs), gases, pH, temperature, and so on.^86^

**Fig. 12:**
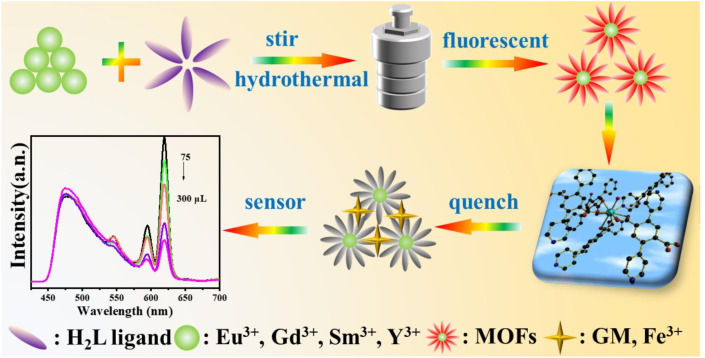
Synthesis and sensing map of 3D RE-MOFs. Reproduced from ref. 87 with permission from Elsevier, copyright 2024.

Tang *et al.* prepared four structurally novel RE-MOFs by hydrothermal method, confirmed their structures by single-crystal X-ray diffraction, and characterized their physicochemical properties by TG, PXRD and FTIR. Finally, the sensing properties were investigated and the possible mechanisms were hypothesized, see Fig. 12.^87^ The successful implementation of this project plays an active role in the application of RE-MOFs in environmental protection and human health detection.

It has been shown that RE-MOFs have the potential to be applied as thin-film active layers in electrochemical sensors for the detection of hydrogen sulfide (H_2_S) at room temperature. This unique fcu-based topology (RE-fcu-MOF) has a very high sensitivity for the detection of H_2_S at concentrations as low as 100 ppb, with a lower detection limit of about 5 ppb.^88^ In addition, carbendazim (CBZ) poses a serious threat to human health due to food-borne residues, and a smartphone-assisted fluorescent sensor based on a bimetallic organic framework (Eu/Tb-MOF) can be constructed. Eu^3+^ and Tb^3+^ emit characteristic red and green light, respectively, through ligand-sensitized luminescence, and the ratio of the fluorescence intensities of the two constitutes the internal reference signal eliminates environmental interference and enables rapid visualization of CBZ in food products.^89^ Dichlorophenol (Dcp) is known for its resistance to degradation as a highly toxic environmental pollutant, and La-MOF can be used to create novel electrochemical sensors for the detection of Dcp due to its large specific surface area and abundance of electrocatalytically active sites.^90^ Wang *et al.* synthesized a pH-stabilized three-dimensional Tb-MOF (JXUST-19) used as a rare switch and blue-shifted MOF sensor for benzaldehyde and salicylaldehyde.^91^ Guo *et al.* reported the preparation of a dual-emitting material, Eu–Ca-MOF, which has excellent photoluminescence properties and can be used as a ratiometric fluorescence sensor (*I*_381_/*I*_590_) for sensitive detection of Hg^2+^ ions.^92^ Xiao *et al.* synthesized a multi-responsive metal–organic framework (MOF), formulated as [Zn(tpbpc)_2_] solvent which can detect Hg^2+^, CrO_4_^2−^ and Cr_2_O_7_^2−^ ions and realize the visual detection of Hg^2+^.^93^

#### 4.1.3 Environmental remediation

As multifunctional soil conditioners, RE-MOFs can enhance soil porosity and permeability through their multistage pore structure, while providing a bionic habitat interface for microbial communities and promoting the growth of soil microorganisms. In addition, the slow-release effect of rare earth ions in RE-MOFs can regulate plant physiological processes: rare earth metal frameworks can slowly release rare earth metal ions, La^3+^ promotes nitrogen uptake by activating nitrate reductase, and Ce^3+^ enhances the electron transfer efficiency of the chloroplast photosystem II, which results in higher photosynthetic rate, drought tolerance, and biomass of the crop, respectively. More importantly, RE-MOFs are able to adsorb heavy metal ions in the soil by physical adsorption or chemical adsorption, in which the metal ions can undergo an ion exchange reaction with heavy metal ions or adsorb heavy metal ions to the surface of the framework by electrostatic gravity, thus reducing the concentration and mobility of heavy metal ions in the soil and reducing their harm to the soil ecosystem and crops.

For example, *Bacillus anthracis*, as a Gram-positive aerobic bacillus, has some ability to survive in the natural environment. Soil can be one of the reservoirs of *Bacillus anthracis* spores. When infected animal carcasses are not handled properly, the spores can penetrate deep into the soil and become enriched in specific environments, threatening ecological security.^94^ To address this problem, metal ions in RE-MOFs may react with chemical groups on the surface of *B. anthracis*, and specific functional groups of proteins, polysaccharides, and other components of the cell wall may undergo coordination or ion exchange reactions to form chemical bonds, thus realizing the adsorption and fluorescence detection of *B. anthracis*.^95^

In summary, RE-MOFs can optimize soil physical properties, strengthen nutrient cycling and synergize the management of compound pollution through the three-in-one mechanism of “structural improvement-biological activation-pollution blocking and controlling”, which has the dual functions of environmental remediation and agricultural efficiency enhancement, and provides a new material paradigm for sustainable soil management.

### 4.2 Pollutant removal

#### 4.2.1 Sewage treatment

**Fig. 13:**
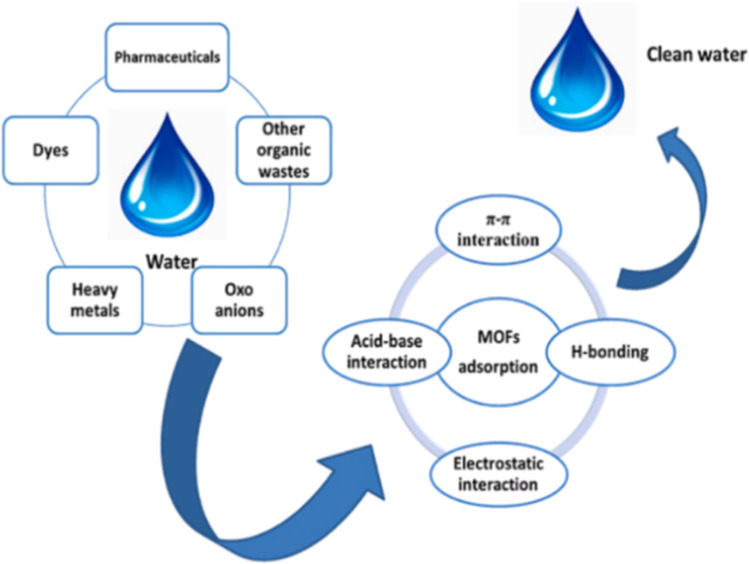
Mechanism of removal of heavy metal ions, dyes and other harmful substances from water by RE-MOFs. Reproduced from ref. 96 with permission from Elsevier, copyright 2022.

Eutrophication of water bodies has become a major challenge for global water environment management, and its core causative factor is the explosive proliferation of algae triggered by excessive input of nutrients such as nitrogen and phosphorus. However, traditional adsorption materials have problems such as insufficient specific surface area and single active site, which make it difficult to realize efficient retention of pollutants. Metal–organic framework materials (MOFs), characterized by high specific surface area, porous structure and abundant functional groups, construct a novel functional platform for the efficient adsorption and removal of pollutants in water bodies.^96^ Rare earth metals (*e.g.*, La^3+^, Ce^3+^) can topologically modify conventional Zn/Cu-MOFs, which can produce significant synergistic effects: the introduction of La^3+^ enhances the adsorption capacity of MIL-101(Cr) for phosphate by 2.3-fold, and the cycling stability of Ce^3+^-doped ZIF-8 was enhanced to 15 cycles, and 92% efficacy was maintained after regeneration. Doping of rare-earth metals effectively improves the performance of monometallic MOFs and provides insights into the study of efficient water purification using RE-MOFs.^97^ The mechanism of removing heavy metal ions, dyes and other harmful substances from water by rare earth MOF materials is shown in Fig. 13.^96^

**Fig. 14:**
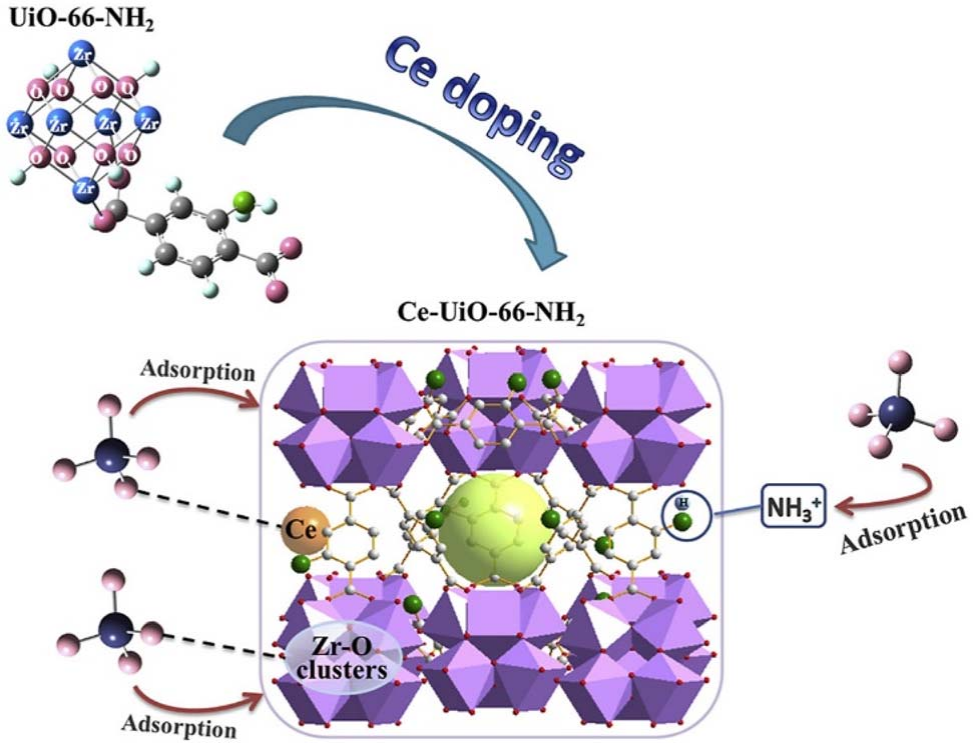
A possible mechanistic model for phosphate trapping on 0.75Ce-UiO-66-NH_2_. Reproduced from ref. 99 with permission from Elsevier, copyright 2020.

As a typical variable rare earth element, cerium (Ce^3+^/Ce^4+^) shows significant advantages in the field of environmental adsorption due to its unique electronic structure ([Xe]4f^1^5d^1^6s^2^) and physicochemical properties. Ce has a relatively small ionic radius, ionic potential, and high affinity for surface hydroxyl groups. Ce has a relatively small ionic radius, ionic potential, and high affinity for surface hydroxyl groups, so Ce doping into the MOF crystal structure will form coordination defects, which provide more active adsorption sites for adsorption of phosphates and arsenates.^98^ Currently, Ce-MOFs-based adsorbents have been successfully applied to the remediation of eutrophic lake sediments, and their retention of trace arsenate (<10 ppb) can reach more than 90%, which provides a new strategy for the targeted treatment of heavy metal-like metal-contaminated waters. Liu *et al.* attempted to use microwave-assisted Ce doping in the pristine crystal structure of UiO-66-NH_2_ to create defects. The 0.75Ce-UiO-66-NH_2_ material absorbed up to 211.86 mg g^−1^ of phosphorus, which exceeded that of the undoped Ce-doped UiO-66-NH_2_, and the model of the phosphorus trapping mechanism of this material is shown in Fig. 14.^99^

The equilibrium adsorption capacity of Ce^3+^ introduced with UiO-67 as a carrier was enhanced by 85% compared to the undoped system,^100^ which was mainly attributed to the electronic modulation of rare earth ions. In contrast, the modification mechanism of MIL-88(Fe) by Ce doping presents different features: more ligand defects and higher Lewis acidity can be introduced into the backbone through a simple Ce doping strategy, which results in the generation of more surface-adsorbed hydroxyl radicals (·OH_ads_) and reactive oxygen species such as single-linear oxygen species (^1^O_2_) and the enhancement of ozone conversion catalytic activity.^100^

Secondly, various pollutants are discharged into water bodies from different sources, resulting in increased water pollution.^101^ Among them, due to the illegal use of organophosphorus pesticides in agricultural production, they are present in water bodies in the form of residues,^102^ which are highly toxic and carcinogenic to humans due to their persistence, bioaccumulation and stability,^103^ and various organophosphorus pesticides can be detected in environmental water, food crops and soils, with widely varying concentrations over time and location, despite the implementation of strict pesticide regulations.^104,105^ Therefore, there is an urgent need to develop novel materials to remove organophosphorus pesticides to protect the environment and human health.^102^

Although RE-MOFs, as emerging environmental functional materials, provide revolutionary solutions for the management of pollutants in water bodies, they still have potential limitations. For example, except for La, Ce, and Nd doping, other rare earth elements have rarely been investigated in adsorption frameworks. Meanwhile, most RE-MOFs materials exhibit poor performance after multiple recycling, which limits their practical applications, and the coordination through organic matter or substrate metal ions may lead to the inactivation of free acidic metal sites in RE-MOFs. Future research should focus on the above aspects in order to promote RE-MOFs from theoretical innovation to engineering application, and provide sustainable technical support for global water environment management.

#### 4.2.2 Air purification

RE-MOFs have been used to study gas adsorption, separation and purification processes.^106^ These potential applications rely on their porous nature, permanent porosity, surface area and pore volume, tunable surface functionality, chemical functionality of organic joints, accessibility of metal sites, and inter-permeability. Meanwhile, the tunable coordination nanospace of MOFs offers amenability to crystal engineering design principles, an aspect that enables MOFs to outperform most traditional adsorbents.^107^

RE-MOFs with zeolite-like topologies maintain backbone stability even after dehydration, and their dynamic structural changes can induce selective adsorption properties. The Eu^3+^, Tb^3+^, and Y^3+^-based fcu-MOF metal–organic skeleton constructed by Xue *et al.* using a reticulated chemical approach achieved specific recognition and truncation effects on sorbate pollutant molecules by modulating the selective entry of the contracted pores.^108^ Such pore size engineering endows the materials with unique molecular sieving functions: on the one hand, the domain-limited pores can realize selective adsorption of volatile organic compounds (VOCs) such as formaldehyde and benzene, and efficiently differentiate and separate the coexisting polar/non-polar molecules in the air by using the kinetic separation property; on the other hand, the rare-earth nodes in the rigid skeleton stabilize the adsorbent by strong coordination, and maintain high VOCs capturing efficiency even under dynamic airflow conditions, the design provides a structural paradigm for the development of smart air purification materials.

In the catalytic treatment of VOCs, Ln-MOFs as catalysts or carriers can significantly enhance the reaction activity and selectivity through the electron modulation effect of rare earth ions (*e.g.*, La^3+^, Ce^3+^). It has been shown that the presence of rare-earth-doped MOFs can enhance the activity and selectivity of the catalysts and promote the reaction of VOCs with oxygen, which can be converted into harmless substances such as CO_2_ and H_2_O at lower temperatures.^109^ In addition, some Ln-MOFs can generate electron–hole pairs under visible light excitation, which leads to the generation of reactive oxygen species such as hydroxyl radicals (·OH) and superoxide radicals (·O_2_^−^), which can decompose the VOCs by the photocatalytic oxidation pathway,^110^ and improve the degradation efficiency. The unique electronic structure and optical properties of rare-earth metal ions make Ln-MOFs potentially applicable in the photocatalytic degradation of VOCs, and Ln-MOFs can also be synergized with other technologies or materials to improve the treatment effect on VOCs. For example, by combining Ln-MOFs with adsorbents and catalysts, the integrated adsorption-catalytic treatment of VOCs can be realized, which can improve the treatment efficiency and reduce the treatment cost.

However, although RE-MOFs show a broad application prospect in the field of gas adsorption, separation, and purification, their actual industrialization process is still limited by multiple technological bottlenecks. From the perspective of synthetic chemistry, the current preparation strategies of some RE-MOFs are in conflict with the principles of green chemistry, such as their fabrication consumes a large amount of energy,^111^ which restricts their potential for large-scale applications. Future research needs to focus on low-carbon preparation techniques, combined with topology modulation and rare earth element recycling strategies, to promote the sustainable transformation of RE-MOFs from the laboratory to industrial scenarios.

## 5. Applications in the medical field

### 5.1 Drug delivery

Drug delivery systems (DDS) are technological systems that comprehensively regulate the distribution of drugs in an organism in terms of space, time, and dose. The development of DDS has made it possible to introduce therapeutic substances into the body through appropriately formulated devices, and to improve efficiency and safety by controlling the rate, time, and location of drug release in the body.^112^ To date, several classes of DDSs, such as micelles,^113^ polymers,^114^ liposomes,^115^ carbon nanomaterials,^116^ gold nanostructures,^117^ bio ceramics,^118^ mesoporous silica^119–121^ and MOFs for cancer therapy.^122,123^ However these DDS have limitations in cancer therapy, to overcome these barriers we can use smart drug delivery systems (SDDS) with innovative nanocarriers, RE-MOF is considered to be one of the representative categories of innovative porous smart nano biomaterials.^124^

#### 5.1.1 Targeted therapies

With their outstanding high specific surface area and porous structure, RE-MOFs present great potential as drug carriers and show unique advantages in drug delivery systems.^15,125^

In the field of anticancer, the paclitaxel loading system constructed on the basis of Eu-MOF is able to achieve precise drug release in the micro-acidic environment of tumors (pH about 5.5–6.5) through pH-responsive release mechanism. This intelligent delivery system can not only increase the effective concentration of the drug by 2–3 times, but also reduce the toxicity of the system by about 40%, which significantly enhances the efficacy of anticancer treatment.^126^

In antimicrobial therapy, rare-earth MOF materials can be used as carriers for the antimicrobial drug vancomycin^127^ to achieve uniform dispersion and slow release of the drug and to improve the effectiveness of local or systemic anti-infective therapy. Zhang *et al.* found that the lanthanide salt-based constructed MOF and two ligands reacted solvent-free at mild temperatures to form ternary lanthanide nanoscale CPs at the 10 gram level.^128^ The *in vitro* antimicrobial activity of these ternary hybrids was investigated using zone of inhibition method, minimum inhibitory concentration, minimum bactericidal concentration and transmission electron microscopy and was found to have excellent antimicrobial properties. The *in vitro* antitumor activity was carried out by measuring the absorbance values by CCK-8 (Cell Counting Kit-8). This simple synthetic method has the potential to produce ternary lanthanide CPs on a large scale at room temperature, which may be promising candidates as antimicrobial compounds and antitumor agents.

Through precise design and modification, the rare-earth MOF materials are able to realize controlled release and targeted delivery of drugs, thus prolonging the duration of drug efficacy, reducing side effects, and increasing the cumulative concentration of drugs in the lesion site. At the same time, Eu-MOF materials show good compatibility in biological systems,^15^ which reduces adverse reactions to the organism and ensures the safety and stability of drug carriers *in vivo*.

#### 5.1.2 Diagnostic imaging

Some rare-earth MOF materials, such as Y-BTCs and Eu-doped Y-BTCs MOFs, can be used as fluorescent markers for *in vivo* imaging due to their luminescent properties. Combining drugs with these luminescent rare-earth MOFs realizes the integration of drug delivery and imaging, which provides a more comprehensive information support for accurate diagnosis and treatment of diseases.^129^ Re-MOFs also show great potential for application in diagnostic imaging due to their unique structure and properties.^130^ The following are innovative applications of Re-MOFs in optical imaging, fluorescence imaging,^131^ magnetic resonance imaging (MRI), and multimodal imaging.

Re-MOFs are ideal for optical imaging due to their excellent optical properties and high stability. Especially as fluorescent markers, Re-MOFs can realize highly sensitive and selective imaging of specific targets *in vivo*, providing an important basis for precise diagnosis of diseases. Francesca Lo Presti *et al.* used ultrasound (US) as a promising synthesis method to produce Y-BTC and Eu-doped Y-BTC MOFs with excellent structural stability and luminescence properties, and demonstrated their potential application in diagnostic imaging,^129^ which provides a new way of thinking about the application of Re-MOFs in fluorescence imaging.

In magnetic resonance imaging, RE-MOFs can significantly enhance the contrast of MRI signals by introducing paramagnetic rare-earth ions such as gadolinium to be used as MRI contrast agents,^130^ which in turn enhances the tissue differences in the images, enabling physicians to more accurately identify lesion areas, thus improving the accuracy of the disease diagnosis and early detection rate. Compared with conventional MRI contrast agents, RE-MOFs contrast agents have higher stability and lower toxicity, taking gadolinium ions as an example, RE-MOFs contrast agent reduces the toxicity of free gadolinium ions by virtue of its high porosity and large specific surface area, which ensures efficient loading of therapeutic agents and makes it more suitable for clinical applications.

Flunitrazepam is one of the most potent prescription psychotropic drugs in the benzodiazepine family.^132^ It is soluble in alcohol, colorless and odorless,^133^ and when combined with alcohol causes medical symptoms such as unconsciousness in the drinker.^134^ Flunitrazepam has been used maliciously in major criminal cases, especially in drug-related sexual assaults against females, posing a serious threat to public safety and social stability.^135–137^ Eu-MOF can be used as a highly selective and sensitive luminescent probe for the detection of flunitrazepam, and has shown excellent detection performance in methanol solutions, alcoholic beverages and urine samples.^134^

Notably, the rare-earth MOF material also enables multimodal imaging capabilities, combining a variety of imaging techniques into one,^138^ Wei *et al.* prepared CsLu_2_F_7_:Yb/Er/Tm-based nanoparticles for CT/UCL dual-modality imaging based on the high X-ray absorption coefficient of Lu.^3^ The dual modality of CT and optical imaging realizes real-time imaging and precise diagnosis of brain tumors. It provides more comprehensive information for accurate diagnosis of diseases. This multimodal imaging technology can fully utilize the advantages of various imaging technologies to improve the accuracy and reliability of diagnosis.

### 5.2 Bioassay

#### 5.2.1 Biosensors

With the rapid development of biomedical technology, biosensors, as an important analytical tool, play a key role in the fields of disease diagnosis^139^ and food safety. In recent years, RE-MOFs have shown a wide range of applications in the field of biosensors.

Rare earth composite MOF has high specific surface area, rich pore structure and excellent physicochemical properties, which enable it to be applied as an ideal sensing element in the construction of biosensors.^140^ Under specific conditions, rare-earth MOFs can specifically recognize and capture target biomolecules, such as proteins, nucleic acids, and small-molecule metabolites,^141^ thus realizing high-sensitivity and high-selectivity biosensing.

**Fig. 15:**
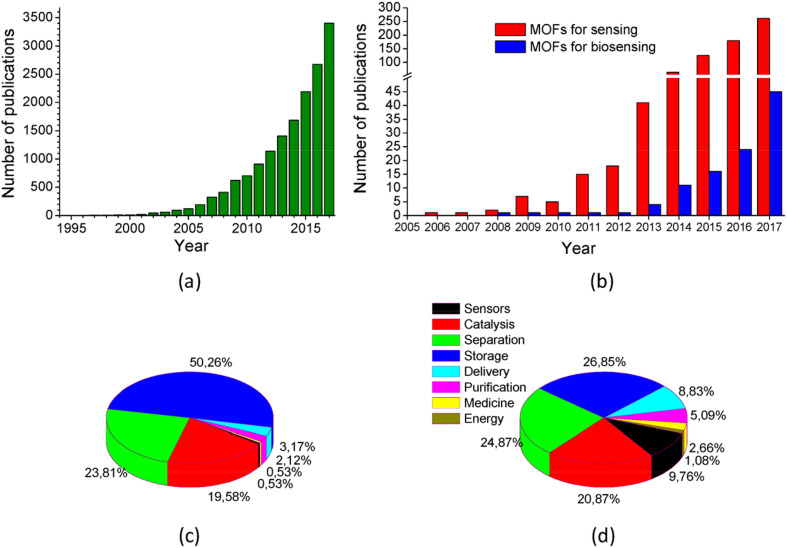
Number of publications of: (a) MOFs in the period 1994–2017; (b) MOFs used for chemical sensor or biosensor development in the period 2005–2017. Distribution of MOF publications in different fields for the years: (c) 2007; and (d) 2017. Source: Web of Science. Reproduced from ref. 140 with permission from MDPI, copyright 2018.

In addition, the unique fluorescence properties of rare earth elements also provide new ideas for the application of rare earth MOFs in biosensors. Eu^3+^-MOF, as a fluorescent sensing element, is able to eliminate the interference of the background fluorescence by using time-resolved fluorescence technology to realize ultra-sensitive detection of biomolecules.^142^ This fluorescence sensing technology is not only suitable for *in vitro* analysis, but can also be extended to *in vivo* imaging, providing powerful support for early diagnosis of diseases and monitoring of treatment (Fig. 15).

#### 5.2.2 Cellular imaging

The unique fluorescence properties of rare earth elements, such as long fluorescence lifetimes and high quantum yields, enable RE-MOFs to eliminate the interference of background fluorescence in bioimaging,^118^ improve signal-to-noise ratios, and achieve high sensitivity and high resolution imaging.^138^ This multimodal imaging technique not only improves diagnostic accuracy, but also provides an important basis for efficacy assessment during disease treatment.

The application of RE-MOFs in bioimaging is also reflected in the targeted recognition and imaging of specific biomolecules. Through surface modification or functionalized design, RE-MOFs can specifically bind to target biomolecules, such as receptors on the surface of tumor cells, biomarkers, *etc.*, to achieve highly selective imaging detection.^141^ This targeted imaging technology provides strong support for early diagnosis and precise treatment of diseases.

### 5.3 Organizational engineering

#### 5.3.1 Bracket material

RE-MOFs, as functional materials, can be used to build or enhance the performance of scaffold materials. By precisely controlling the pore size, morphology and surface properties of MOFs, scaffold materials can be designed to match specific tissue regeneration needs.^143^ In addition, rare earth elements have unique bioactivities in living organisms, which can promote cell proliferation and angiogenesis,^144^ further enhancing the advantages of rare earth composite MOFs as scaffold materials for applications.

Functionally, the stent material has a relatively single function, but the introduction of the rare earth composite material MOF can give the stent antibacterial, anti-inflammatory, cell growth promotion and other functions. Determination of antibacterial activity of RE-MOF [Y_2_(MH)_6_]_*n*_-DMF (1), [Er_2_(MH)_6_]_*n*_ (2), [Yb_2_(MH)_6_]_*n*_ (3) and [La(MH)_3_]_*n*_ (4) against *Escherichia coli* and *Staphylococcus aureus* by Zhu *et al.*^145^ The results showed that the antimicrobial drugs derived from RE-MOFs not only possessed stronger antimicrobial activities, but also exhibited excellent stability and long-lasting antimicrobial effects. In addition, rare earth elements have unique bioactivities in living organisms and can promote cell proliferation and angiogenesis, further enhancing the advantages of rare earth composite MOFs as scaffold materials.

Conventional stent materials may induce a stronger immune response or risk of toxicity due to insufficient biocompatibility, which in turn affects cell growth and tissue regeneration. RE-MOFs exhibit good bio inertness or bioactivity,^144^ which can reduce the occurrence of foreign body reactions and inflammation, and ensure that the scaffold materials are stable *in vivo* for a long period of time. Meanwhile, through surface modification and biofunctionalized design, the biocompatibility of rare-earth composite MOFs can be further improved to promote the interaction between cells and scaffold materials and accelerate the tissue regeneration process.

In summary, the rare earth composite MOF, as a biocompatible scaffold material with good biocompatibility, shows a broad application prospect in the field of tissue regeneration. Its unique structure and excellent properties provide new ideas and methods for the development of tissue engineering. In the future, with the in-depth study of the properties of RE-MOFs and their innovative applications in biocompatible scaffold materials, it is believed that they will provide strong support for the realization of efficient and precise tissue regeneration.

## 6. Conclusion and outlook

Rare-earth composite metal–organic framework materials (RE-MOFs) have realized breakthrough applications in energy, environment and healthcare fields by virtue of their unique ligand tunability, porous structure and multi-functional optical/electrical/magnetic properties of rare-earth elements. In the field of energy, the synergistic effect of rare earth ions and organic ligands significantly improves the efficiency of photocatalytic hydrogen production, CO_2_ reduction, and electrochemical energy storage; in the field of environmental treatment, the high specific surface area and selective adsorption capacity provide an efficient platform for VOCs capture, fluorescence sensing, and catalytic degradation of ozone; and in the direction of biomedicine, the synergistic effect of the luminescent properties of rare earth elements and the drug-loading advantages of metal–organic frameworks (MOFs) has given rise to new diagnostic and therapeutic platforms, such as precision targeted diagnostics, high-resolution imaging and controlled drug delivery systems. Future research needs to focus on three major dimensions: first, deepen the material design theory, combine machine learning and high-throughput computing to precisely regulate the rare-earth coordination microenvironment, and develop smart materials with dynamic stability (*e.g.*, humidity/heat and acid/alkali resistance) and directional functions (*e.g.*, optical/magnetic response); second, promote cross-scale integration and application, and explore the composite strategy of RE-MOFs with flexible devices, nano-enzymes, or biofilms to build a “detection–purification–repair” platform. Third, to break through the bottleneck of industrialization, reduce the preparation cost through green synthesis technology (mechanochemical method, continuous flow synthesis), and systematically evaluate the risk of rare earth ion migration and develop biodegradable materials, so as to balance the advantages of performance and ecological safety. With the deepening of multidisciplinary crossover, RE-MOFs are expected to move from the “functional exploration” in the laboratory to the “precise regulation” in practical scenarios, and become a transformative solution for global challenges such as carbon neutralization, environmental remediation, and precision medicine, *etc.*, and the core breakthroughs may depend on the development of rare earth-ligands, which can be utilized in the production of rare earths. The core breakthrough may depend on the atomic level analysis of the electron transfer mechanism at the rare earth-ligand interface and the innovative construction of dynamically responsive material systems.

## Conflicts of interest

There are no conflicts to declare.

## Data Availability

No primary research results, software or code have been included and no new data were generated or analysed as part of this review.
